# Cerebellar dysfunction in frontotemporal dementia: intra-cerebellar pathology and cerebellar network degeneration

**DOI:** 10.1007/s00415-025-13046-8

**Published:** 2025-03-25

**Authors:** Jana Kleinerova, Marlene Tahedl, Mary Clare McKenna, Angela Garcia-Gallardo, Siobhan Hutchinson, Orla Hardiman, Cédric Raoul, Fabrice Ango, Bernard Schneider, Pierre-Francois Pradat, Ee Ling Tan, Peter Bede

**Affiliations:** 1 Computational Neuroimaging Group (CNG), School of Medicine, Trinity College Dublin, Room 5.43, Pearse Street, Dublin 2, Ireland; 2 Department of Neurology, St James’s Hospital, Dublin, Ireland; 3 ALS Reference Centre, University of Montpellier, CHU Montpellier, Montpellier, France; 4 INM (Neuroscience Institute of Montpellier), University of Montpellier, INSERM, CNRS, Montpellier, France; 5 Bertarelli Platform for Gene Therapy, École Polytechnique Fédérale de Lausanne (EPFL), Geneva, Switzerland; 6 Biomedical Imaging Laboratory, CNRS, INSERM, Sorbonne University, Paris, France; 7 Department of Neurology, Pitié-Salpêtrière University Hospital, Paris, France

**Keywords:** Amyotrophic lateral sclerosis, Cerebellum, Motor neuron disease, Frontotemporal dementia, Neuroimaging, Magnetic resonance imaging

## Abstract

**Background:**

Amyotrophic lateral sclerosis (ALS) and frontotemporal dementia (FTD) share overlapping clinical, genetic, and neuroimaging features; a spectrum of conditions commonly referred to as the ALS-FTD continuum. The majority of imaging studies focus on supratentorial pathology, and phenotype-defining motor, cognitive, and behavioural profiles are often exclusively attributed to supratentorial degeneration overlooking the contribution of cerebellar pathology.

**Methods:**

A multimodal neuroimaging study was conducted to evaluate phenotype-associated cerebello-cerebral connectivity profiles in ALS-FTD, behavioural variant frontotemporal dementia (bvFTD), non-fluent variant (nfvPPA), and semantic variant primary progressive aphasia (svPPA). Structural connectivity, functional connectivity, and volumetric analyses were conducted.

**Results:**

Radial diffusivity analyses detected impaired bilateral cerebello-frontal, cerebello-parietal, and cerebello-temporal connectivity in all study groups along the ALS-FTD spectrum. Cerebello-occipital disconnection was captured in ALS-FTD and nfvPPA. Spinocerebellar disconnection was detected in *C9orf72* negative ALS-FTD and nfvPPA. *C9orf72* positive ALS-FTD patients exhibited both anterior and posterior lobe cerebellar volume loss, while bvFTD and nfvPPA patients showed posterior cerebellar atrophy. Flocculonodular degeneration was observed in nfvPPA and cerebellar crura atrophy in bvFTD. Bilateral corticospinal tract and corpus callosum degeneration was detected in ALS-FTD, bvFTD, and nfvPPA. Primary motor cortex volume reductions were captured in both ALS-FTD and nfvPPA.

**Conclusions:**

Our analyses capture significant cerebro-cerebellar disconnection in frontotemporal dementia. Corticospinal tract and motor cortex degeneration can be readily detected in non-ALS phenotypes. Intra-cerebellar pathology, coupled with the degeneration of cerebellar projections and the ensuing dysfunction of cerebro-cerebellar networks likely contribute to phenotype-defining clinical profiles in frontotemporal dementia. Infratentorial disease burden and cerebellar network dysfunction should, therefore, be carefully considered in FTD, and phenotype-defining neuropsychological profiles should not be solely attributed to supratentorial degeneration.

## Introduction

Amyotrophic lateral sclerosis (ALS) and frontotemporal dementia (FTD) form part of the same disease spectrum with shared clinical, imaging, and genetic features [1–3]. There are distinct clinical phenotypes along the ALS-FTD continuum with distinctive neuroimaging patterns [4–6], but ALS also often presents with comorbid FTD [7, 8]. From a radiological standpoint, ALS is classically associated with preferential motor cortex, corpus callosum, brainstem, and spinal cord degeneration [9–11], whereas FTD is primarily linked to phenotype- and genotype-associated patterns of frontotemporal degeneration [12, 13]. ALS with comorbid FTD (ALS-FTD) exhibits both the neuroimaging features of ALS and FTD with widespread grey and white matter degeneration [1, 7]. More recently, extensive basal ganglia and thalamic degeneration have also been described in the ALS-FTD spectrum and linked to cognitive, behavioural, and extrapyramidal manifestations [14–17]. One of the shared genetic underpinnings of ALS and FTD are GGGGCC hexanucleotide repeat expansion in *C9orf72* which may manifest in either ALS, FTD, or comorbid ALS-FTD [2, 18]. While early studies of ALS linked *C9orf72* status to particularly severe extra-motor disease burden [19–21], later studies confirmed that severe frontotemporal and subcortical involvement is not unique to GGGGCC hexanucleotide repeat expansion carriers [14, 22, 23]. One of the relatively overlooked aspects of the radiological profile of ALS-FTD is the degree of infratentorial involvement. As ALS is dominated by UMN and LMN dysfunction and FTD is dominated by phenotype-defining neuropsychological deficits, it is not surprising that early imaging studies have largely focused on cerebral, i.e. supratentorial aspects of neurodegeneration. After sporadic reports of cerebellar pathology in ALS [24, 25] and reports of cerebellar disease burden in FTD [4, 12], dedicated cerebellar imaging studies have been increasing confirming that infratentorial pathology is an important feature of the conditions along the ALS-FTD spectrum.

While emotional, cognitive, behavioural, and psychiatric manifestations [26–31] of cerebellar pathology are well characterised in the literature, cognitive aspects of cerebellar function are often under-recognised in the clinical setting, and severe neuropsychological deficits are typically exclusively attributed to supratentorial pathology [32]. Observations from stroke have consistently linked cognitive processes to the posterior cerebellar lobe [26, 27], and recent neuroimaging studies have mapped specific cognitive processes to lobules VI, VIIA, VIIB, IX, and crus I/II [33–35]. The cerebellar substrates of language impairment [36], pseudobulbar affect [29, 37, 38], disinhibition [39], deficits in social cognition [40], and impulsivity [39] are also well described. Despite the wealth of lesion studies and academic studies of cerebellar physiology, cerebellar dysfunction in ALS-FTD is notoriously under-evaluated [4, 12, 25, 41–45], as the majority of imaging studies in both ALS and FTD continue to solely focus on supratentorial disease burden patterns [3]. Studies that do focus on infratentorial pathology in ALS-FTD typically evaluate atrophy and grey matter disease primarily, whilst intra-cerebellar white matter alterations and the degeneration of deep-cerebellar nuclei are under-evaluated [24, 25, 46]. It is increasingly clear that cerebellar pathology contributes significantly to phenotype-defining neuropsychological manifestations, and core clinical profiles are not exclusively driven by supratentorial changes. The biggest gap in the literature, however, is not the paucity of cerebellar studies in ALS-FTD [32], but studies specifically evaluating cerebellar projections [44]. Emerging data from ALS suggest that cerebellar pathology does not occur in isolation, but with the degeneration of cerebellar afferents and efferents, there is a progressive disconnection of the cerebellum from other brain regions. Accordingly, the main objective of this study is the systematic characterisation of structural and functional cerebellar connectivity with specific brain regions in distinct clinical phenotypes along the ALS-FTD spectrum. We hypothesise that irrespective of the degree of cerebellar atrophy, there are considerable alterations in cerebellar connectivity, the severity of which varies in specific disease entities. Ultimately, we seek to assess and confirm our hypothesis that network-level, cerebellar circuitry dysfunction underpins clinical phenotypes in ALS-FTD.

## Methods

A prospective, single-centre, multimodal imaging study was conducted with a uniform neuroimaging protocol. A total of 198 participants were included; 29 sporadic *C9orf72* negative patients with ALS-FTD (ALS-FTD C9NEG), 24 ALS-FTD patients with GGGGCC hexanucleotide repeat expansions in *C9orf72* (ALS-FTD C9POS), 10 patients with behavioural variant frontotemporal dementia (bvFTD), 15 patients with non-fluent variant primary progressive aphasia (nfvPPA), 7 patients with semantic variant primary progressive aphasia (svPPA), and 113 healthy controls. Exclusion criteria for all participants included prior brain surgery, cerebrovascular events, traumatic brain injury, demyelination, neoplastic, paraneoplastic, or autoimmune disorders. In addition, healthy controls had no neurological, neurosurgical, or psychiatric diagnoses and no first- or second-degree relatives with neurodegenerative disorders. All invited participants were screened for MRI safety (pacemakers, aneurysm clips, claustrophobia, etc.) prior to recruitment. Patients with incidentally identified intracranial findings, such as arachnoid cysts, hydrocephalus, pineal cysts, or meningiomas, were excluded from the analyses. All participants gave informed consent prior to study inclusion.

### Ethics approval

The research protocol was approved by Medical Research (Ethics) Committee of Beaumont Hospital Dublin (REC reference: 08/90), and all participants gave informed consent to participate.

### Participants

Basic demographic variables including sex, age at image acquisition, handedness, level of education, and years of education were carefully recorded. Complementary information regarding alcohol and smoking history, occupation was also documented. Accompanying clinical information with regards to symptom duration, date of diagnosis, family history of neurological disease, current medications were also noted. Patients with ALS were diagnosed according to the EL Escorial criteria and have been screened for a panel [47] of ALS-associated genetic variants (*ALS2, ANG, ATXN2, CHCHD10, CHMP2B, DAO, DCTN1, ELP3, ERBB4, FIG4, FUS, HNRNPA1, MATR3, NEFH, NEK1, OPTN, PFN1, PRPH, SARM1, SETX, SIGMAR1, SOD1, SPAST, SPG11, SQSTM1, TAF15, TARDBP, TBK1, UNC13A, UBQLN2, VAPB,* and *VCP)* as well as GGGGCC hexanucleotide repeat expansions in *C9orf72*. Methods for genetic screening have been described previously [43, 48] Motor disability of patients with ALS has been assessed by the revised ALS functional rating scale (ASLFRS-r). Patients with FTD were diagnosed according to the Rascovsky criteria [49, 50]. All patients had cognitive screening with the Edinburgh Cognitive and Behavioural ALS Screen (ECAS), which is a validated multi-domain assessment scale that accounts for motor disability in patients with ALS [51, 52].

### Neuroimaging

A uniform, purpose-designed imaging protocol was implanted on a 3 T Philips Achieva MRI platform which included T1-weighted (T1w) imaging, fluid-attenuated inversion recovery (FLAIR), diffusion-weighted imaging (dMRI), and resting-state functional MRI (rs-fMRI). A 3D Inversion Recovery prepared Spoiled Gradient Recalled echo (IR-SPGR) sequence was utilised to acquire T1w images with the following parameters: 160 sagittal slices with no interslice gap, flip angle (FA) = 8°, VR = 1 mm^3^, SENSE factor = 1.5. TR/TE = 8.5/3.9 ms, TI = 1060 ms, FOV of 256 × 256 × 160 mm. FLAIR images were acquired axially using an Inversion Recovery Turbo Spin Echo (IR-TSE) sequence with a repetition time (TR)/echo time (TE) = 11,000/125 ms, inversion time (TI) = 2800 ms, field of view (FOV) = 230 × 183 × 150 mm, voxel resolution (VR) = 0.65 × 0.87 × 4 mm. A spin-echo echo-planar imaging (SE-EPI) pulse-sequence was utilised to acquire dMRI data with a 32-direction Stejskal–Tanner diffusion encoding scheme, dynamic stabilisation, and spectral presaturation with inversion recovery (SPIR) fat suppression: TR/TE = 7639/59 ms, FOV = 245 × 245 × 150 mm, 60 axial slices with no interslice gaps, FA = 90°, VR = 2.5 mm^3^, SENSE factor = 2.5. An echo-planar imaging (EPI) sequence was used to acquire 220 volumes of resting-state functional MRI (rs-fMRI) data to evaluate blood-oxygen-level-dependent (BOLD) signal fluctuations at rest with eyes closed with the following parameters: TR/TE = 2000/35 ms, FOV = 233 × 233 × 120 mm, VR = 2.875 mm × 2.875 mm × 4 mmm, SENSE factor = 2.5. As per our ethics approval and quality control (QC) procedures, FLAIR and T1-weighted images of each participant were individually reviewed for unexpected incidental intracranial findings before the inclusion of in the research study. Based on this, two individuals with large arachnoid cysts and two participants with meningiomas were excluded.

### Morphometric grey matter analyses

Cerebellar volume alterations were assessed using the Computational Anatomy Toolbox (CAT12) [53]. Standard pre-processing procedures were followed including denoising, affine registration, partial volume segmentation, skull-stripping, and spatial normalisation. Infratentorial regions-of-interest (ROIs) were defined as per the SUIT cerebellar parcellation scheme [54]: (1) anterior lobe (SUIT labels I-V), (2) posterior lobe (SUIT labels VI-IX), (3) flocculonodular lobe (SUIT label X), (4) crura (merged SUIT labels Crura I and II), and (5) vermis (SUIT label “Vermis”). The cerebellar nuclei were defined in accordance to the Julich Brain Cytoarchitectonic Atlas [55]: (1) dorsal dentate, (2) ventral dentate, (3) interposed, and (4) fastigial. The “Brodmann 4a” label of the Anatomy3 atlas [56] was used to define the primary motor cortex (M1). The CAT12 pipeline was also utilised for total intracranial volume (TIV) estimations.

### Structural connectivity analyses

**Fig. 1:**
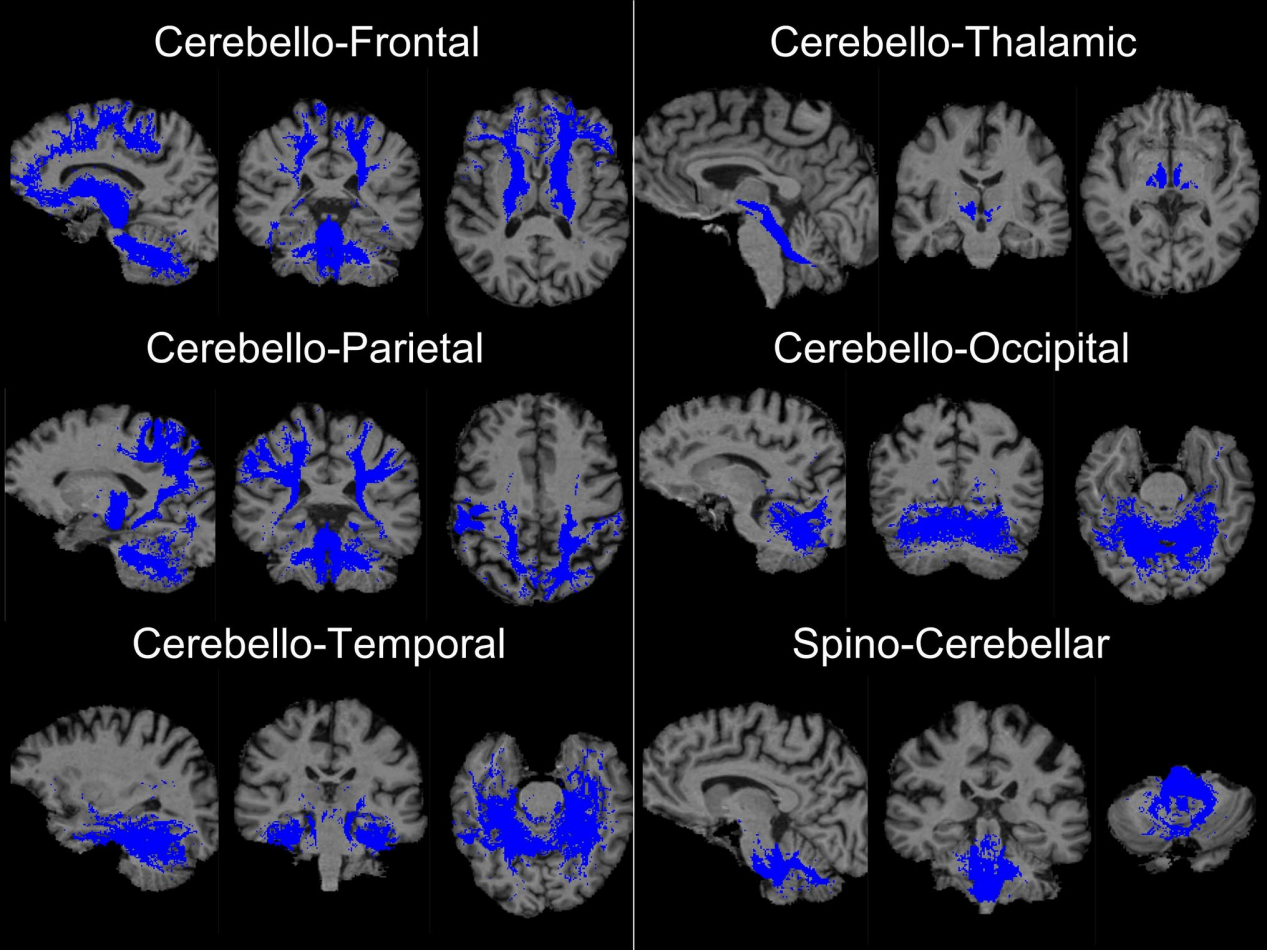
Representative tractography outputs of cerebellar projections. Representative sagittal, coronal, and axial views are shown for each tract

The integrity of cerebellar white matter projections was assessed by probabilistic tractography. Raw dMRI data were pre-processed using *MRtrix3* [57]. Standard pre-processing steps were used; denoising, Gibb’s ringing artifact removal, motion-, eddy current-, and bias field-corrections. Voxelwise fibre orientation distribution (fODF) was estimated using constrained spherical deconvolution (CSD) method [58] before normalisation [59]. The benefit of implementing CSD compared to other tensor-based models is that it evaluates crossing fibres more accurately even at low *b*-values. The following white matter projections were evaluated by tractography: (1) cerebello-frontal, (2) cerebello-parietal, (3) cerebello-temporal, (4), cerebello-occipital, (5) cerebello-thalamic, and (6) spinocerebellar, i.e. inferior brainstem-to-cerebellum. Cerebellar masks were defined using merged SUIT labels, cerebral ROI masks defined based on Automated Anatomical Labeling (AAL) atlas labels, and the inferior brainstem label of the Hammers atlas [60] was used to define for ‘spinocerebellar’ tractography. The cerebral segment of the corticospinal tracts (CST) was tracked from the primary motor cortex (M1) to the brainstem in each hemisphere separately. Inter-hemispheric transcallosal fibres were mapped between the right and left primary motor cortices. Probabilistic tractography [61] was run with 5000 streamlines for each tract. An illustrative example of cerebellar projections is shown in Fig. 1. The track-density imaging (TDI) method was used [62] to map track data onto to a high-resolution image in native space and binarised to generate maps for subsequent average fractional anisotropy (FA) and radial diffusivity (RD) value extraction in each tract.

### Functional analyses

Analogous to the structural connectivity analyses, blood-oxygen-level-dependent (BOLD) signal associations were explored between the cerebellum and above described ROIs. Moreover, functional connectivity was also evaluated along the corticospinal tracts (CST) between the medulla oblongata and motor cortex (M1) as well as along the mid-body of the corpus callosum (CC) between the primary motor cortices in the left and right hemispheres. Raw input fMRI data were pre-processed using the FEAT package of the FMRIB Software Library (FSL) [63]. Brain extraction, intensity normalisation, and slice-time corrections were undertaken. Head-motion artifacts were corrected using FSL’s ICA-based Automatic Removal of Motion Artifacts (ICA-AROMA) [64]. The pre-processed data were registered to MNI152 2 mm standard space implementing an initial linear co-registration of the native functional images to native high-resolution T1w data using 6 degrees of freedom (DOFs), followed by non-linearly warping to standard space using 12 DOFs. Analogous to our structural connectivity analyses, (1) cerebello-frontal, (2) cerebello-parietal, (3) cerebello-temporal, (4), cerebello-occipital, (5) cerebello-thalamic, (6) spinocerebellar, (7) CST, and (8) transcallosal functional connectivity was evaluated. FC was estimated using the CoSMoMVPA toolbox in Matlab R2022b (The Mathworks, Natick, USA) as Fisher *z*-transformed Pearson correlation analyses between the mean BOLD time course of each pair of ROIs.

### Statistical modelling

RStudio (version 2022.12.0 + 353; R version 4.2.2) was used for statistical analyses. Welch two-sample *t* tests were performed to test differences in age and years of education between all patients merged and healthy controls (HC). Chi-squared tests were performed to test differences in sex and handedness frequencies between all patients and healthy controls. One-way analysis of variance (ANOVA) was used to evaluate differences in neuroimaging metrics between patient groups and HC, correcting for the confounding effects of age, sex, handedness, and years of education. In our volumetric analyses, total intracranial volume (TIV) was also introduced as a covariate. The main effect “Study group” was evaluated in the ANOVA omnibus test, and if this was significant, post hoc testing was performed to detect relevant pairwise contrasts using Tukey’s HSD tests. Tukey’s HSD test outputs are corrected for multiple comparisons and indicated as *p*_adj_ in Table 2.

### Data availability

Additional information on data acquisition, processing pipelines can be requested from the corresponding author. Clinical information, genetic results, and neuroimaging data from individual patients cannot be made available due to departmental policies.

## Results

Following MRI screening for incidental intracranial findings MRI data from 198 participants were systematically analysed stratified according to the main diagnosis into ALS-FTD C9NEG, ALS-FTD C9POS, bvFTD, nfvPPA, svPPA, and HC groups. The demographic profiles and group comparisons are presented in Table 1. All patients with ALS-FTD tested negative for the above panel [47] of ALS-associated genetic variants and were stratified into “ALS-FTD C9NEG” and “ALS-FTD C9POS” groups based on their *C9orf72* carrier status.

**Table 1:** The demographic profile of study participants

	ALS-FTD C9NEG	ALS-FTD C9POS	bvFTD	nfvPPA	svPPA	All patients	HC	*T* test* [W]* Chi-squared *[C*^*2*^*]*
Total number of subjects	29	24	10	15	7	85	113	n.a
Complete T1w datasets	29	24	10	15	7	85	113	n.a
Complete dMRI datasets	29	24	10	14	6	83	113	n.a
Complete fMRI datasets	26	22	7	8	2	65	111	n.a
Age [y, mean ± SD]	63.66 ± 11.13	55.50 ± 9.05	63.40 ± 6.43	71.33 ± 6.82	68.57 ± 6.29	63.08 ± 11.54	59.36 ± 10.48	*W: t*(182.6) = 2.45, *p* = 0.015
Sex, F/M	7/22	9/15	4/6	9/6	4/3	33/52	57/56	*C*^*2*^*: X*^*2*^(1, *N* = 198) = 2.19, *p* = .139
Handedness, R/L	26/3	20/4	10/0	15/0	7/0	78/7	106/7	*C*^*2*^*: X*^*2*^(1, *N* = 198) = 0.075, *p* = 0.784
Years of education [y, mean ± SD]	13.45 ± 4.21	14.04 ± 3.51	12.20 ± 4.18	13.00 ± 2.65	15.86 ± 2.19	13.59 ± 3.67	14.68 ± 3.52	*W: t*(177.0) = −2.11, *p* = 0.036

ALS: amyotrophic lateral sclerosis, ALS-FTD C9NEG: sporadic, *C9orf72* negative patients with ALS-FTD, ALS-FTD C9POS: ALS-FTD patients with GGGGCC hexanucleotide repeat expansions *C9orf72*, bvFTD: behavioural variant frontotemporal dementia, dMRI: diffusion-weighted MRI, F: female, fMRI: functional MRI, FTD: frontotemporal dementia, HC: healthy control, L: left-handed, M: male, MRI: magnetic resonance imaging, N: sample size, nfvPPA: non-fluent variant primary progressive aphasia, n.a.: not applicable, R: right-handed, SD: standard deviation, svPPA: semantic variant primary progressive aphasia, y: years

### Structural connectivity

**Fig. 2:**
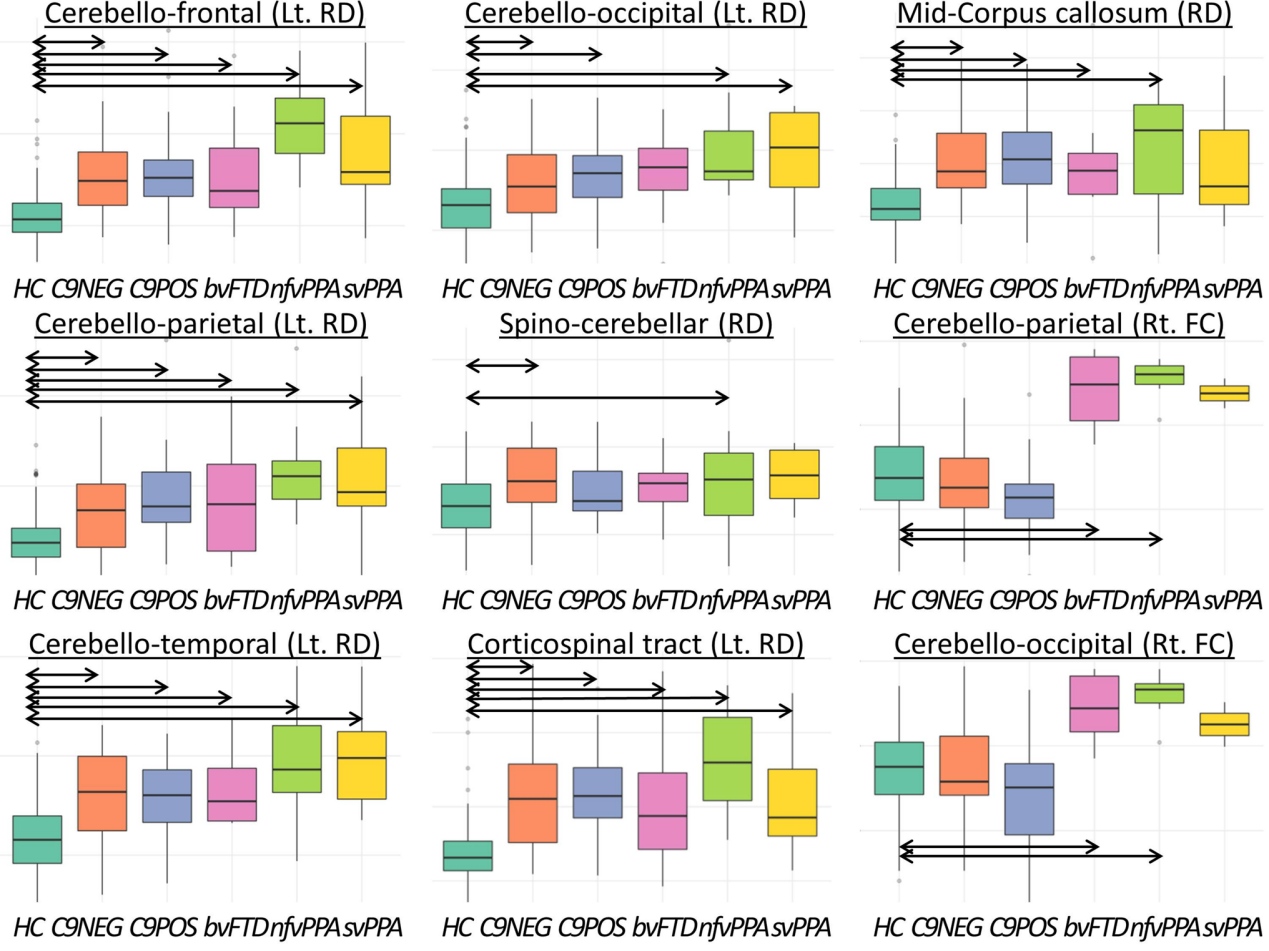
Structural and functional connectivity profiles in the study groups. *bvFTD* behavioural variant frontotemporal dementia (pink colour), *C9NEG* sporadic, *C9orf72* negative patients with ALS-FTD (orange colour), *C9POS* ALS-FTD patients with GGGGCC hexanucleotide repeat expansions *C9orf72* (blue colour)*, FC* Functional connectivity, *HC* healthy controls (turquoise colour), *Lt* Left hemisphere, *nfvPPA* non-fluent variant primary progressive aphasia (neon green colour), *RD* Radial diffusivity as a proxy of “structural connectivity” *Rt* Right hemisphere, *svPPA* semantic variant primary progressive aphasia (yellow colour). Only representative contrasts are shown in one hemisphere and only radial diffusivity profiles are shown for diffusivity analyses; full statistical details are provided in Table 2 which presents statistics for all imaging metrics in both hemispheres. Significant differences compared to controls are highlighted by horizontal arrows

As outlined in detail in Table 2, radial diffusivity (RD) analyses capture bilateral cerebello-frontal, cerebello-parietal, and cerebello-temporal alterations in all patient groups compared to healthy controls. The ALS-FTD C9NEG, ALS-FTD C9POS, and nfvPPA also exhibit bilateral cerebello-occipital disconnection. Among the patient cohorts, nfvPPA is often the worst affected, followed by ALS-FTD C9POS and ALS-FTD C9NEG. RD analyses have also revealed spinocerebellar tract degeneration in ALS-FTD C9NEG and nfvPPA. Fractional anisotropy is less sensitive in detecting these changes; nonetheless, FA has also detected bilateral cerebello-frontal disconnection in ALS-FTD C9POS and nfvPPA and right hemispheric alterations in ALS-FTD C9NEG. Contrary to RD, however, FA did capture increased bilateral cerebello-thalamic connection in the ALS-FTD C9NEG group and in the right hemisphere in nfvPPA. With regards to non-cerebellar projections, corticospinal tract degeneration has been readily detected in all study groups based on RD, which is bilateral with the exception of the svPPA where only left hemispheric changes were noted. FA analyses capture bilateral CST degeneration in ALS-FTD C9NEG, ALS-FTD C9POS, and nfvPPA (Table 2). Transcallosal commissural degeneration was detected in ALS-FTD C9NEG, ALS-FTD C9POS, bvFTD, and nfvPPA based on RD and in the two ALS-FTD groups based on FA (Fig. 2).

**Table 2:** The neuroimaging profiles of study groups and their post hoc pairwise contrasts

	One-way ANOVA	Post hoc testing		
	Left hemisphere		Right hemisphere	
	*F* value (DOF), *p* value	*p* value Significant pairwise contrasts (post hoc)	*F* value (DOF), *p* value	*p* value Significant pairwise contrasts (post hoc)
Connectivity: cerebello-frontal				
SC: RD	*F*(2,157) = 37.10, *p* < 0.001*	- C9NEG – HC [*p*_adj_ < 0.001] - C9POS – HC [*p*_adj_ < 0.001] - bvFTD – HC [*p*_adj_ = 0.005] - nfvPPA – HC [*p*_adj_ < 0.001] - svPPA – HC [*p*_adj_ < 0.001] - nfvPPA-C9NEG [*p*_adj_ = 0.003] - nfvPPA-C9POS [*p*_adj_ = 0.006] - nfvPPA-bvFTD [*p*_adj_ = 0.033]	*F*(2,157) = 41.31, *p* < 0.001*	- C9NEG – HC [*p*_adj_ < 0.001] - C9POS – HC [*p*_adj_ < 0.001] - bvFTD – HC [*p*_adj_ = 0.005] - nfvPPA – HC [*p*_adj_ < 0.001] - svPPA – HC [*p*_adj_ = 0.007]
SC: FA	*F*(2,157) = 10.94, *p* < 0.001*	- C9POS – HC [*p*_adj_ = 0.003] - nfvPPA – HC [*p*_adj_ < 0.001]	*F*(2,157) = 13.65, *p* < 0.001*	- C9NEG – HC [*p*_adj_ = 0.046] - C9POS – HC [*p*_adj_ < 0.001] - nfvPPA – HC [*p*_adj_ = 0.004]
FC	*F*(2,150) = 0.163, *p* = 0.850	*n.a*	*F*(2,150) = 1.23, *p* = 0.296	n.a
Connectivity: cerebello-parietal				
SC: RD	*F*(3,315) = 23.48, *p* < 0.001*	- C9NEG – HC [*p*_adj_ = 0.002] - C9POS – HC [*p*_adj_ < 0.001] - bvFTD – HC [*p*_adj_ = 0.028] - nfvPPA – HC [*p*_adj_ < 0.001] - svPPA – HC [*p*_adj_ = 0.003] - nfvPPA-C9NEG [*p*_adj_ = 0.017]	*F*(2,157) = 29.48, *p* < 0.001*	- C9NEG – HC [*p*_adj_ < 0.001] - C9POS – HC [*p*_adj_ < 0.001] - bvFTD – HC [*p*_adj_ = 0.003] - nfvPPA – HC [*p*_adj_ < 0.001] - svPPA – HC [*p*_adj_ = 0.021]
SC: FA	*F*(2,157) = 4.21, *p* = 0.017*	n.a	*F*(2,157) = 4.73, *p* = 0.010*	n.a
FC	*F*(2,150) = 1.27, *p* = 0.285	n.a	*F*(2,150) = 4.16, *p* = 0.018*	- bvFTD – HC [*p*_adj_ < 0.001] - nfvPPA – HC [*p*_adj_ < 0.001] - bvFTD – C9NEG [*p*_adj_ < 0.001] - nfvPPA – C9NEG [*p*_adj_ < 0.001] - svPPA – C9NEG [*p*_adj_ = 0.041] - bvFTD – C9POS [*p*_adj_ < 0.001] - nfvPPA – C9POS [*p*_adj_ < 0.001] - svPPA – C9POS [*p*_adj_ = 0.009]
Connectivity: cerebello-temporal				
SC: RD	*F*(2,157) = 22.36, *p* < 0.001*	- C9NEG – HC [*p*_adj_ < 0.001] - C9POS – HC [*p*_adj_ < 0.001] - bvFTD – HC [*p*_adj_ = 0.028] - nfvPPA – HC [*p*_adj_ < 0.001] - svPPA – HC [*p*_adj_ < 0.001] - nfvPPA-C9NEG [*p*_adj_ = 0.031] - nfvPPA-C9POS [*p*_adj_ = 0.035]	*F*(2,157) = 25.36, *p* < 0.001*	- C9NEG – HC [*p*_adj_ < 0.001] - C9POS – HC [*p*_adj_ < 0.001] - bvFTD – HC [*p*_adj_ < 0.001] - nfvPPA – HC [*p*_adj_ < 0.001] - svPPA – HC [*p*_adj_ < 0.001]
SC: FA	*F*(2,157) = 0.637, *p* = 0.530	n.a	*F*(2,157) = 1.42, *p* = 0.246	n.a
FC	*F*(2,150) = 0.515, *p* = 0.599	n.a	*F*(2,150) = 0.844, *p* = 0.432	n.a
Connectivity: cerebello-occipital				
SC: RD	*F*(2,157) = 11.47, *p* < .001*	- C9NEG – HC [*p*_adj_ = 0.026] - C9POS – HC [*p*_adj_ = 0.010] - nfvPPA – HC [*p*_adj_ < 0.001] - svPPA – HC [*p*_adj_ = 0.028]	*F*(2,157) = 14.91, *p* < 0.001*	- C9NEG – HC [*p*_adj_ = 0.002] - C9POS – HC [*p*_adj_ = 0.007] - bvFTD – HC [*p*_adj_ = 0.012] - nfvPPA – HC [*p*_adj_ < 0.001]
SC: FA	*F*(2,157) = 2.40, *p* = 0.094	n.a	*F*(2,157) = 2.05, *p* = 0.132	n.a
FC	*F*(2,150) = 1.08, *p* = 0.342	n.a	*F*(2,150) = 3.64, *p* = 0.029*	- bvFTD – HC [*p*_adj_ = 0.002] - nfvPPA – HC [*p*_adj_ < 0.001] - bvFTD – C9NEG [*p*_adj_ = 0.010] - nfvPPA – C9NEG [*p*_adj_ < 0.001] - bvFTD – C9POS [*p*_adj_ < 0.001] - nfvPPA – C9POS [*p*_adj_ < 0.001]
Connectivity: cerebello-thalamic				
SC: RD	*F*(2,157) = 0.900, *p* = 0.407	n.a	*F*(2,157) = 2.44, *p* = 0.090	n.a
SC: FA	*F*(2,157) = 4.46, *p* = 0.013*	- C9NEG – HC [*p*_adj_ = 0.025]	*F*(2,157) = 5.59, *p* = 0.005*	- C9NEG – HC [*p*_adj_ = 0.011] - nfvPPA – HC [*p*_adj_ < 0.001] - nfvPPA – C9POS [*p*_adj_ = 0.001]
FC	*F*(2,150) = 2.72, *p* = 0.070	n.a	*F*(2,150) = 1.46, *p* = 0.236	n.a
Connectivity: spinocerebellar				
SC: RD	*F*(2,157) = 6.67, *p* = 0.002*	- C9NEG – HC [*p*_adj_ = 0.009] - nfvPPA – HC [*p*_adj_ = 0.032]		
SC: FA	*F*(2,157) = 1.11, *p* = 0.332	n.a		
FC	*F*(2,142) = 0.926, *p* = 0.399	n.a		
Connectivity: corticospinal				
SC: RD	*F*(2,157) = 45.21, *p* < 0.001*	- C9NEG – HC [*p*_adj_ < 0.001] - C9POS – HC [*p*_adj_ < 0.001] - bvFTD – HC [*p*_adj_ = 0.005] - nfvPPA – HC [*p*_adj_ < 0.001] - svPPA – HC [*p*_adj_ = 0.029]	*F*(2,157) = 46.40, *p* < 0.001*	- C9NEG – HC [*p*_adj_ < 0.001] - C9POS – HC [*p*_adj_ < 0.001] - bvFTD – HC [*p*_adj_ = 0.001] - nfvPPA – HC [*p*_adj_ < 0.001]
SC: FA	*F*(2,157) = 18.74, *p* < 0.001*	- C9NEG – HC [*p*_adj_ = 0.009] - C9POS – HC [*p*_adj_ < 0.001] - nfvPPA – HC [*p*_adj_ < 0.001]	*F*(2,157) = 19.72, *p* < 0.001*	- C9NEG – HC [*p*_adj_ = 0.022] - C9POS – HC [*p*_adj_ < 0.001] - nfvPPA – HC [*p*_adj_ < 0.001]
FC	*F*(2,142) = 0.085, *p* = 0.919	n.a	*F*(2,142) = 1.33, *p* = 0.268	*n.a*
Connectivity: mid-corpus callosum				
SC: RD	*F*(2,157) = 37.41, *p* < 0.001*	- C9NEG – HC [*p*_adj_ < 0.001] - C9POS – HC [*p*_adj_ < 0.001] - bvFTD – HC [*p*_adj_ = 0.024] - nfvPPA – HC [*p*_adj_ < 0.001]		
SC: FA	*F*(2,157) = 12.03, *p* < 0.001*	- C9NEG – HC [*p*_adj_ = 0.048] - C9POS – HC [*p*_adj_ = 0.005]		
FC	*F*(2,142) = 1.29, *p* = 0.278	n.a		
Volumetry: cerebellar cortex				
Anterior cerebellar lobe			Posterior cerebellar lobe	
	*F*(2,157) = 5.51, *p* < 0.001*	- C9POS – HC [*p*_adj_ = 0.033]	*F*(2,157) = 5.79, *p* = 0.004*	- C9POS – HC [*p*_adj_ = 0.030] - bvFTD – HC [*p*_adj_ = 0.037] - nfvPPA – HC [*p*_adj_ = 0.030]
Flocculonodular lobe			Cerebellar crura	
	*F*(2,157) = 3.74, *p* = 0.026*	- nfvPPA – HC [*p*_adj_ = 0.004]	*F*(2,157) = 4.79, *p* = 0.001*	- bvFTD – HC [*p*_adj_ = 0.031]
Cerebellar vermis				
	*F*(2,157) = 2.656, *p* = *0.0734*	n.a		
Volumetry: cerebellar nuclei				
Dorsal dentate nucleus			Ventral dentate nucleus	
	*F*(2,157) = 3.66, *p* = 0.028*	n.a	*F*(2,157) = 1.72, *p* = 0.183	n.a
Interposed nucleus			Fastigial nucleus	
	*F*(2,157) = 2.20, *p* = 0.114	n.a	*F*(2,157) = 0.182, *p* = 0.834	n.a
Volumetry: motor cortex				
M1				
	*F*(2,157) = 40.75, *p* < 0.001*	- C9NEG – HC [*p*_adj_ < 0.001] - C9POS – HC [*p*_adj_ < 0.001] - nfvPPA – HC [*p*_adj_ < 0.001]		

*adj* adjusted, *ANOVA* analysis of variance, *bvFTD* behavioural variant frontotemporal dementia, *C9NEG* sporadic, *C9orf72* negative patients with ALS-FTD, *C9POS* ALS-FTD patients with GGGGCC hexanucleotide repeat expansions *C9orf72*, *DOF* degrees of freedom, *dMRI* diffusion-weighted magnetic resonance imaging, *FA* fractional anisotropy, *FC* functional connectivity, *FTD* frontotemporal dementia, *HC* healthy control, *HSD* honest significant difference, *M1* primary motor cortex, *n.a.* not applicable, *nfvPPA* non-fluent variant primary progressive aphasia, *RD* radial diffusivity, *rs-fMRI* resting-state functional magnetic resonance imaging, *SC* structural connectivity, *svPPA* semantic variant primary progressive aphasia, *T1w* T1-weighted

^*^Significant at an alpha-level of *p* ≤ 0.05

### Functional connectivity

As presented in Table 2, functional connectivity analyses captured limited dissociation between the ROIs. Increased cerebello-parietal and cerebello-occipital functional connectivity was captured in the right hemisphere in bvFTD and nfvPPA compared to healthy controls as well as in contrast to the two ALS-FTD groups (Fig. 2). Somewhat unexpectedly, no commissural (transcallosal) or pyramidal FC alterations were detected in either study group.

### Volumetrics

**Fig. 3:**
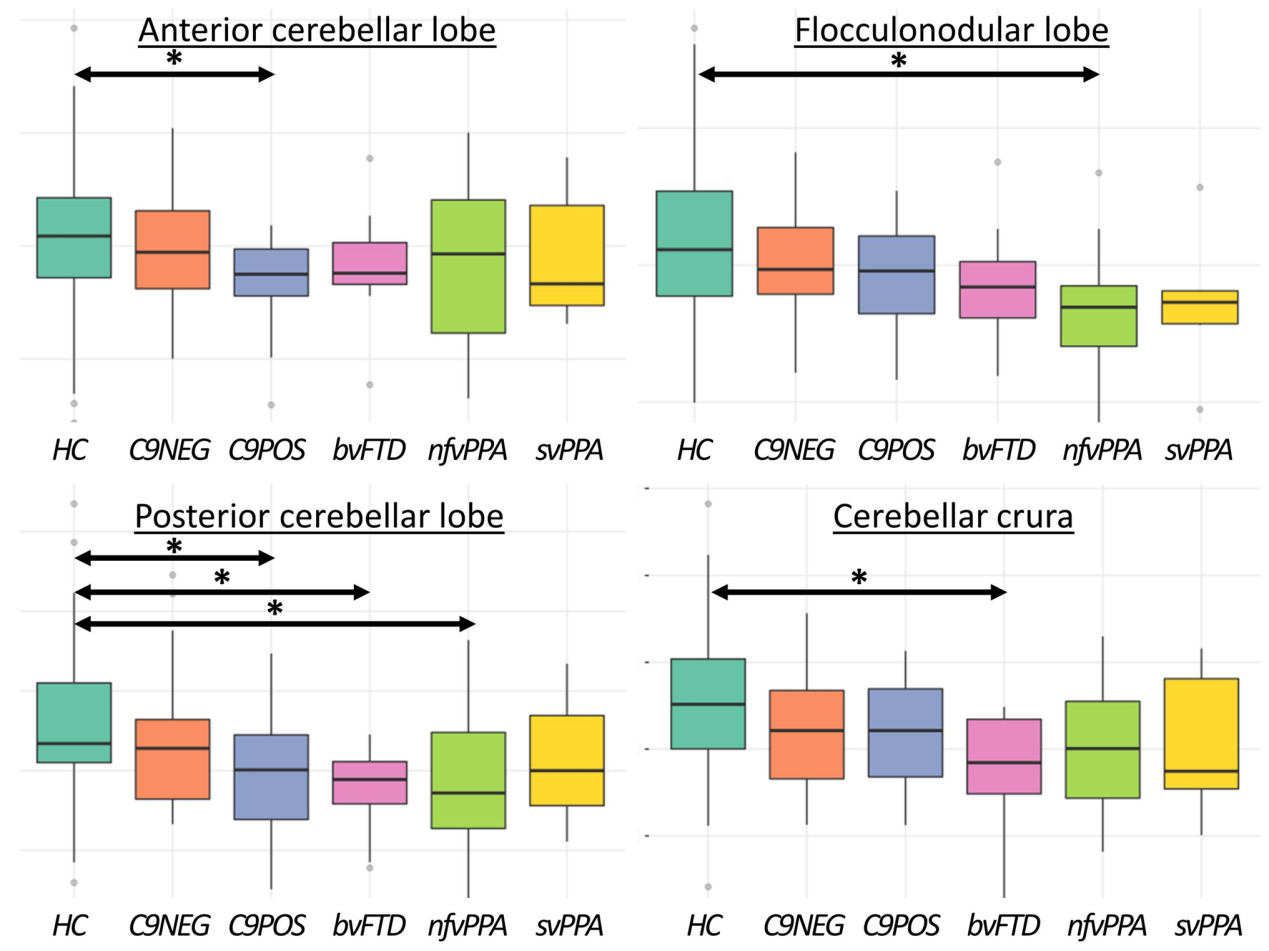
The volumetric profiles of the study groups. *bvFTD* behavioural variant frontotemporal dementia (pink colour), *C9NEG* sporadic, *C9orf72* negative patients with ALS-FTD (orange colour), *C9POS* ALS-FTD patients with GGGGCC hexanucleotide repeat expansions *C9orf72* (blue colour)*, HC* healthy controls (turquoise colour), *nfvPPA* non-fluent variant primary progressive aphasia (neon green colour), *svPPA* semantic variant primary progressive aphasia (yellow colour). Significant differences compared to controls are highlighted by horizontal arrows with asterisks

As shown in Table 2, ALS-FTD C9POS patients exhibited anterior cerebellar volume loss. ALS-FTD C9POS, bvFTD, and nfvPPA patients showed posterior cerebellar atrophy. The nfvPPA cohort succumbs to flocculonodular degeneration and bvFTD patients exhibit cerebellar crura atrophy compared to controls (Table 2 & Fig. 3). The vermis is not significantly affected in either cohort. The evaluated deep-cerebellar nuclei (ventral and dorsal dentate, interposed, and fastigial nuclei) did not demonstrate significant volume loss in either patient group compared to controls. Primary motor cortex volume reductions were captured in both ALS-FTD groups as well as in the nfvPPA cohort compared to healthy controls (Fig. 3).

## Discussion

Our analyses capture significant cerebro-cerebellar disconnection in ALS-FTD. Bilateral cerebello-frontal, cerebello-parietal, and cerebello-temporal connectivity is impaired in all patient groups along the ALS-FTD spectrum and cerebello-occipital disconnection was also captured in ALS-FTD C9NEG, ALS-FTD C9POS, and nfvPPA. Phenotype-associated patterns of cerebellar atrophy were detected; *C9orf72* positive ALS-FTD patients exhibiting anterior lobe atrophy, bvFTD, and nfvPPA patients showing preferential posterior lobe volume loss, and patients with bvFTD demonstrating cerebellar crura degeneration and flocculonodular atrophy was observed in nfvPPA. Interestingly, pyramidal tract, corpus callosum, and primary motor cortex degeneration was observed in non-ALS-FTD phenotypes; bilateral corticospinal tract and corpus callosum degeneration was captured in both bvFTD and nfvPPA and primary motor cortex volume reductions in nfvPPA. Moreover, spinocerebellar disconnection was detected in *C9orf72* negative ALS-FTD and nfvPPA confirming that not only the cerebro-cerebellar projections are affected but also cerebellar afferents conveying important proprioceptive and cutaneous information.

### Cerebellar dysfunction in ALS-FTD

Cerebellar dysfunction is notoriously under-recognised in both ALS and FTD [4, 12, 25, 65, 66], partly because supratentorial disease burden predominates clinical manifestations and partly because of the challenges associated with detecting, measuring, and tracking progressive cerebellar changes in vivo. While cerebellar pathology is often simplistically linked to impaired coordination and balance, it contributes to a wealth of clinically relevant manifestations in ALS, such as eye-movement abnormalities, impaired dexterity, dysarthria, dysphagia, gait impairment, pseudobulbar affect, and altered respiratory patterns, clinical manifestations that are often exclusively linked to cerebral and brainstem pathology [29, 37, 67]. Cerebellum-mediated cognitive processes are particularly overlooked in ALS, and to a lesser extent, in FTD as well, two conditions where cognitive deficits are typically attributed to supratentorial changes alone. Cognitive deficits arising from posterior cerebellar lobe pathology, such as verbal memory impairment, visuospatial, executive, social cognition, or language deficits are seldom considered [26, 27, 33–36, 40] Vermis-associated emotional dysregulation, impulsivity, and irritability [39] are rarely considered. Previous FTD studies indicate particularly severe cerebellar degeneration in ALS-FTD and bvFTD, and more selective cerebellar involvement in language variant FTDs [42, 66, 68]. Most cerebellar imaging studies exclusively evaluate grey matter changes and metabolic, functional, and white matter profiles are surprisingly understudied [45, 69, 70]. Similar to the paucity of dedicated cerebellar imaging studies in FTD, there are relatively few post-mortem studies specifically commenting on infratentorial changes. These describe ubiquitin and p62-positive neuronal cytoplasmic inclusions that were noted in the granular layer of the cerebellar cortex [71–73]. Compared to the significant cerebellar degeneration in *C9orf72*, cerebellar atrophy may be less obvious in *MAPT* [72, 74].

### Clinical implications

The considerable spinocerebellar tract pathology detected in *C9orf72* negative ALS-FTD and nfvPPA and the anterior cerebellar lobe atrophy captured in C9POS ALS-FTD unravel one of the substrates of gait impairment and disequilibrium in these cohorts. Patients with ALS-FTD are known to be at a higher risk of falls, which is typically attributed to motor weakness overlooking coexisting proprioceptive deficits and some degree of sensory ataxia. While spinal cord imaging has a considerable literature in ALS and other motor neuron diseases [9, 75], it is seldom performed in FTD [76]. Spinal studies of ALS primarily focus on cross-sectional area reductions, grey matter atrophy, and pyramidal tract degeneration, and sensory components of cord pathology are relatively overlooked [77]. The intra-cerebellar and cerebro-cerebellar white matter alterations in the ALS-FTD cohorts are likely to impact on the motor aspects of the condition, exacerbating bulbar dysfunction, gait impairment, and declining dexterity [78]. While pseudobulbar affect is typically linked to corticobulbar tract degeneration [79], impaired cerebellar gating has been consistently implicated in the aetiology of this clinical phenomenon [37, 38]. It is noteworthy that severe corticospinal tract degeneration was detected in non-ALS phenotypes (bvfTD, nfvPPA, and svPPA), primary motor cortex volume reductions captured in nfvPPA and that transcallosal commissural degeneration was also detected in bvFTD and nfvPPA. CST and CC degeneration is traditionally regarded as a hallmark of ALS, PLS, and HSP [75, 80], but motor cortex and pyramidal tract degeneration is not classically associated with FTD. While frontotemporal pathology is well recognised in ALS [3], motor system degeneration is less recognised in FTD [81]. In line with our own findings, recent studies highlight the importance of screening for motor impairment in FTD cohorts [82] and keep the risk of transitioning to ALS in mind. Another clinical aspect of cerebellar dysfunction in ALS-FTD is the recognition and screening for domains of cerebellum-mediated cognitive domains. We have identified posterior lobe predominant cerebellar atrophy in ALS-FTD C9POS, bvFTD, and nfvPPA. Posterior lobe cerebellar pathology has been consistently linked to cognitive manifestations [26, 27] with specific functions mapped to specific lobules [33–36].The dissection of cerebral-derived and cerebellar-derived cognitive impairment is particularly challenging in a spectrum of conditions where severe frontotemporal, subcortical, and cerebellar degeneration coexist. Accordingly, the administration of validated neuropsychological batteries that specifically explore cerebellar-mediated cognitive functions should be considered. Such batteries and screening tools have been successfully implemented in HSP and various ataxia syndromes [83]. One of the most important finding of the study is that despite the relatively limited volumetric alterations, very significant white matter changes have been detected in all of the phenotypes along the ALS-FTD spectrum highlighting the biomarker potential of connectivity metrics in ALS-FTD.

### Academic considerations

In our study, the most vulnerable cerebellar tracts were the ones that project to cortical regions preferentially affected in FTD, i.e. frontal, temporal, and parietal lobes. These tracts were affected bilaterally in all of our patient cohorts. This may well be consistent with the notion that inter-connected brain regions show concomitant neurodegeneration [84], and emerging studies demonstrating connectivity-based propagation patterns [85]. These observations shift the focus from cortical vulnerability patterns to connectivity alterations and help to reconceptualise ALS-FTD as a “disconnection syndrome” or “network disease”. Cerebellar changes in FTD have been previously linked to specific genotypes [4, 12, 32]. GGGGCC hexanucleotide expansion status associated with crus I and lobule VIIa degeneration and MAPT with vermis degeneration [12]. Cerebellar atrophy can be captured in presymptomatic *C9orf72* mutation carriers [19, 86]. There has been a prevailing notion that cerebellar and frontotemporal changes are particularly marked in *C9orf72*, but recent studies have demonstrated that severe cerebellar and frontotemporal degeneration is not unique to this genotype [14, 22]. While recent studies unequivocally demonstrate cerebellar dysfunction in ALS [25, 44], there is a lingering notion of putative cerebellar compensatory processes based on increased functional and metabolic activation patterns [25, 87–90]. Our study did capture increased FA in cerebello-thalamic projections in ALS-FTD C9NEG and in the right hemisphere in nfvPPA, but this was not supported by RD or FC alterations. Similarly, increased cerebello-parietal and cerebello-occipital functional connectivity changes were detected in the right hemisphere of bvFTD and nfvPPA patients. Despite these unexpected findings, the vast majority of our analyses reveal compelling evidence of cerebro-cerebellar and spinocerebellar disconnection. We also note that there is no post-mortem evidence to convincingly support adaptive or compensatory processes [87, 91] as no hypertrophic changes were identified in any brain regions in ALS. While adaptive changes have been described decades after stroke, traumatic brain injury and childhood insults such as poliomyelitis [92, 93], there is no compelling evidence of similar processes in ALS. From a methodological perspective, RD clearly outperforms FA in terms of detection sensitivity, which may have practical implications for biomarker development, i.e. the selection of imaging metrics that most readily detect pathological change in vivo. Not only did the functional connectivity analyses detect some counterintuitive trends, FC analyses did not capture commissural (transcallosal) or pyramidal FC alterations despite clear evidence of CST and CC degeneration from the diffusivity analyses. These results would indicate that rs-fMRI and BOLD-derived FC analyses may have relatively limited sensitivity in identifying clinically relevant pathological changes. Ultimately, the benefit of undertaking a prospective multimodal study with multiple complementary imaging modalities is the ability to contrast the performance of a panel of quantitative markers in detecting underlying pathological change. In our case, RD performed better than FA, which in turn performed better than FC in capturing cerebro-cerebellar disconnection. Therefore, there may be limited rationale to include rs-fMRI in clinical and pharmaceutical studies if high-quality dMRI is already part of the protocol.

### Limitations and future directions

This study has a number of limitations. While our data compellingly demonstrate cerebro-cerebellar connectivity alterations along the ALS-FTD spectrum, we acknowledge the cohort size limitation of our study groups. In the absence of sufficient longitudinal data, we have only performed cross-sectional analyses in this study, which only offers a snapshot of dynamic, progressive neurodegenerative processes. We also need to acknowledge, that while our ALS cohorts have undergone thorough genetic testing, only some of the FTD patients were screened for common FTD-associated genetic variants. Another limitation of the study is that we did not explore correlations with neuropsychological data. As these cohorts exhibit both considerable supra- and infra-tentorial degeneration, it would be very difficult to delineate the contribution of cerebral and cerebellar pathology to impairments in specific cognitive domains. The cognitive ramifications of cerebellar degeneration should, therefore, be ideally studied in patient cohorts where there is relatively limited comorbid frontotemporal change, such as patients with primary ataxia syndromes. We also acknowledge that, despite their unique clinical and radiological profiles [94, 95], right-temporal lobe variant a.k.a. semantic behavioural variant FTD patients were not included in this study. Based on the preliminary data presented herein, large, prospective, longitudinal studies are needed to comprehensively characterise the evolution of cerebellar dysfunction in FTD and the neuropsychological ramifications of cerebellar pathology in frontotemporal dementia.

## Conclusions

Patients with frontotemporal dementia do not only exhibit cerebellar disease burden, but cerebello-cerebral circuitry is also severely affected. Cerebellar tracts projecting to phenotype-defining cortical regions are particularly vulnerable. The core clinical manifestations of FTD phenotypes are not merely underpinned by distinct patterns of supratentorial degeneration, but cerebellar dysfunction also contributes to phenotype-defining motor and neuropsychological deficits.

## Abbreviations

AAL: Automated Anatomical Labeling (AAL) atlas
AD: Alzheimer’s disease
ALS: Amyotrophic lateral sclerosis
ALS-FTD: Comorbid ALS and FTD
ALS-FTD C9NEG: Sporadic, *C9orf72* negative patients with ALS-FTD
ALS-FTD C9POS: ALS-FTD patients with GGGGCC hexanucleotide repeat expansions* C9orf72*
ALSod: ALS online database
ANOVA: Analysis of variance (ANOVA)
ASO: Antisense oligonucleotide
bvFTD: Behavioural variant frontotemporal dementia
BOLD: Blood-oxygen-level-dependent (BOLD) signal
*C9orf72*: Chromosome 9 open reading frame 72
CC: Corpus callosum
CI: Confidence interval
CT: Cortical thickness
CSD: Constrained spherical deconvolution
CST: Corticospinal tract
DOFs: Degrees of freedom
DTI: Diffusion tensor imaging
DWI: Diffusion-weighted imaging
EMG: Electromyography
EMM: Estimated marginal mean
EPI: Echo-planar imaging
FA: Fractional anisotropy
FC: Functional connectivity
fMRI: Functional MRI
FLAIR: Fluid-attenuated inversion recovery
fODF: Fibre orientation distribution function
FOV: Field of view
FSL: FMRIB´s Software Library
FTD: Frontotemporal dementia
FTLD: Frontotemporal lobar degeneration
FWE: Familywise error
GM: Gray matter
HARDI: High angular resolution diffusion imaging
HC: Healthy control
HD: Huntington’s disease
HSP: Hereditary spastic paraplegia
ICA-AROMA: Automatic removal of motion artifacts
IR-SPGR: Inversion recovery prepared spoiled gradient recalled echo
IQR: Interquartile range
LAS: Local adaptive segmentation
LH: Left hemisphere
Lt: Left
LMN: Lower motor neuron
M1: Primary motor cortex
ML: Machine-learning
MND: Motor neuron disease
MNI: Montreal Neurological Institute
MNI152: Montreal Neurological Institute 152 standard space
MRI: Magnetic resonance imaging
MRS: MR spectroscopy
nfvPPA: Non-fluent variant primary progressive aphasia
NISALS: Neuroimaging Society in ALS
NIV: Non-invasive ventilation
NODDI: Neurite orientation dispersion and density imaging
*p*_adj_: Adjusted *p*-value
PBA: Pseudobulbar affect
PCR: Polymerase chain reaction
PD: Parkinson’s disease
PMC: Primary motor cortex
QC: Quality control
RH: Right hemisphere
Rt: Right
RD: Radial diffusivity
ROI: Region of interest
rs-fMRI: Resting-state functional MRI
SC: Structural connectivity
SD: Standard deviation
SE-EPI: Spin-echo echo-planar imaging
SENSE: Sensitivity encoding
SPIR: Spectral presaturation with inversion recovery
svPPA: Semantic variant primary progressive aphasia
T: Tesla
T1w: T1-weighted imaging
TCV: Total cerebellar volume
TDI: Track-density imaging
TE: Echo time
TI: Inversion time
TIV: Total intracranial volume
Tukey HSD: Tukey’s honest significant difference
TR: Repetition time
UMN: Upper motor neuron
VR: Voxel resolution
WM: White matter
